# Combined Immunotherapy and Hepatic Resection in Cutaneous Melanoma Liver Metastases

**DOI:** 10.7759/cureus.106374

**Published:** 2026-04-03

**Authors:** Ana Luís Martins, Joana Oliveira, Adriana Ferreira, Rui Caetano Oliveira, Rui Martins

**Affiliations:** 1 General Surgery, Portuguese Institute of Oncology of Coimbra Francisco Gentil, Coimbra, PRT; 2 Oncology, Portuguese Institute of Oncology of Coimbra Francisco Gentil, Coimbra, PRT; 3 Pathology, Coimbra University and Hospital Center, Coimbra, PRT

**Keywords:** immunotherapy, liver metastasis, malignant melanoma, multidisciplinary management, nivolumab

## Abstract

Melanoma is an aggressive malignancy with a high risk of distant metastasis, including hepatic involvement, which is associated with poor prognosis. We report a patient initially diagnosed with stage IA melanoma who developed an isolated liver metastasis seven years later. Molecular analysis identified a TP53 mutation and PD-L1 expression ≥ 1%. Treatment with nivolumab achieved a sustained radiological and biochemical response over four years. Owing to favorable systemic control and resectable disease, surgical liver resection was performed. Follow-up demonstrated no evidence of recurrence. At 48 months after initiation of immunotherapy and 12 months after surgery, the patient remains alive and disease-free. This case supports the integration of immunotherapy and surgery in selected patients with metastatic melanoma to the liver and highlights the importance of multidisciplinary management.

## Introduction

Malignant melanoma is the ninth most common cancer and the second leading cause of cancer-related mortality. In patients with distant metastases, the five-year survival rate is approximately 23%, underscoring that metastatic disease is the primary contributor to melanoma-related deaths [1]. Malignant melanoma can spread hematogenously to any organ, with distant metastases occurring in approximately 30% of patients. However, hepatic involvement is observed in approximately 15% of metastatic cases and is associated with poor prognosis [2,3]. The propensity for widespread dissemination contributes to the highly aggressive and poor prognosis of advanced-stage melanoma [4].

The optimal management of metastatic melanoma remains controversial, particularly regarding the role of locoregional treatments such as metastasectomy [5,6]. Historically, surgical resection has been reserved for highly selected patients with limited disease. However, treatment strategies for advanced melanoma have evolved significantly with the introduction of immune checkpoint inhibitors, including antiprogrammed cell death protein 1 (PD-1) and cytotoxic T-lymphocyte-associated protein 4 (CTLA-4) inhibitors, which have redefined therapeutic approaches.

In this context, the potential role of surgical resection following response to systemic immunotherapy remains an area of ongoing clinical interest. Some studies suggest a possible survival benefit in selected patients with isolated hepatic metastases and controlled extrahepatic disease treated with a combination of systemic therapy and surgery [1].

Here, we report a case of metastatic melanoma to the liver treated with nivolumab, followed by hepatic resection after a sustained response to immunotherapy. This case aims to contribute to the current discussion regarding the role of surgery in selected patients in the immunotherapy era.

## Case presentation

A man in his 40s, with no relevant past medical or surgical history, underwent wide local excision with sentinel lymph node biopsy for a presternal melanoma. Histopathological examination confirmed stage IA melanoma (American Joint Committee on Cancer (AJCC) classification: pT1b, N0(sn), M0), characterized by a Breslow thickness of 0.63 mm, Clark IV invasion, and a high mitotic rate (2 mitoses/mm^2^), without ulceration or lymphovascular invasion. The cervical sentinel lymph node was negative. Subsequent molecular analysis identified a TP53 mutation, with no other mutations detected (BRAF and NRAS wild type). Additionally, the tumor cell proportion score for programmed death-ligand 1 (PD-L1) was ≥1%. After the initial management, the patient underwent a standard clinical follow-up.

Seven years later, he presented to the emergency department with acute right upper quadrant pain and a hemoglobin level of 8 g/dL. Initial abdominal ultrasound revealed a hepatic lesion in the left lobe, raising suspicion of active bleeding. Subsequent angiography demonstrated hemorrhage originating from two nodules supplied by branches of segments II and III of the left hepatic artery. Superselective catheterization of these vessels was performed, followed by successful embolization using microspheres and microcoils, achieving hemostasis without immediate complications. Remaining laboratory tests were within normal limits, except for mildly elevated lactate dehydrogenase levels. Four days after embolization, an ultrasound-guided percutaneous liver biopsy confirmed metastatic melanoma with extensive necrosis.

**Figure 1:**
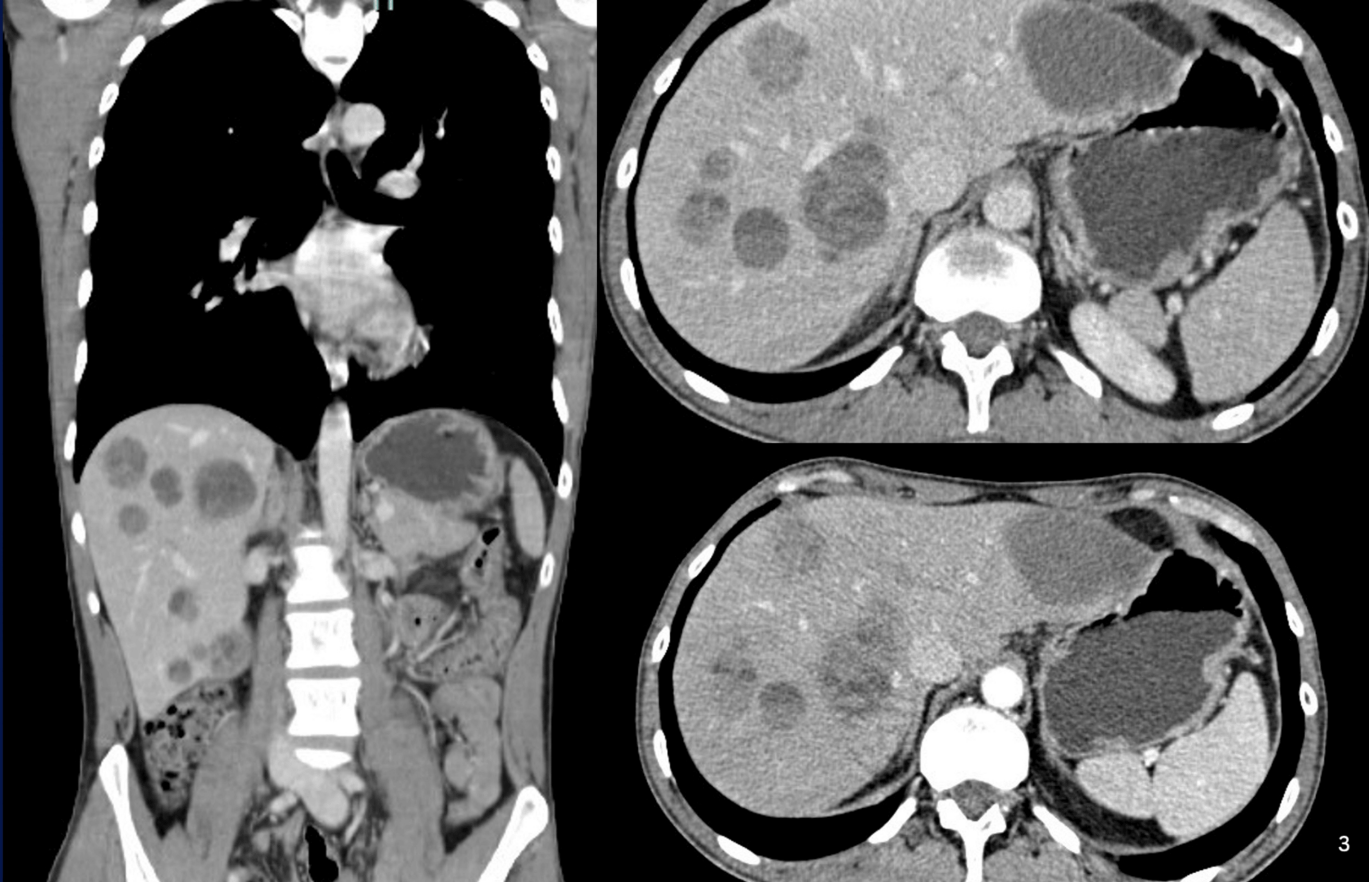
Contrast-enhanced CT imaging showing bilobar hepatic metastases Abdominal CT images (coronal view on the left and axial views on the right) showing a dominant lesion in the left hepatic lobe (segments II–III), measuring approximately 6 cm. Additional smaller lesions are visible, consistent with bilobar hepatic metastases.

Immunohistochemical analysis revealed positivity for HMB-45 and S100 protein, while molecular testing for a BRAF mutation was negative. Staging thoraco-abdominopelvic and brain CT scans revealed no evidence of extrahepatic metastases. The abdominal component is illustrated in Figure 1. Concurrently, tumor markers, such as alpha-fetoprotein, carcinoembryonic antigen, and carbohydrate antigen 19-9, were all within normal limits.

**Figure 2:**
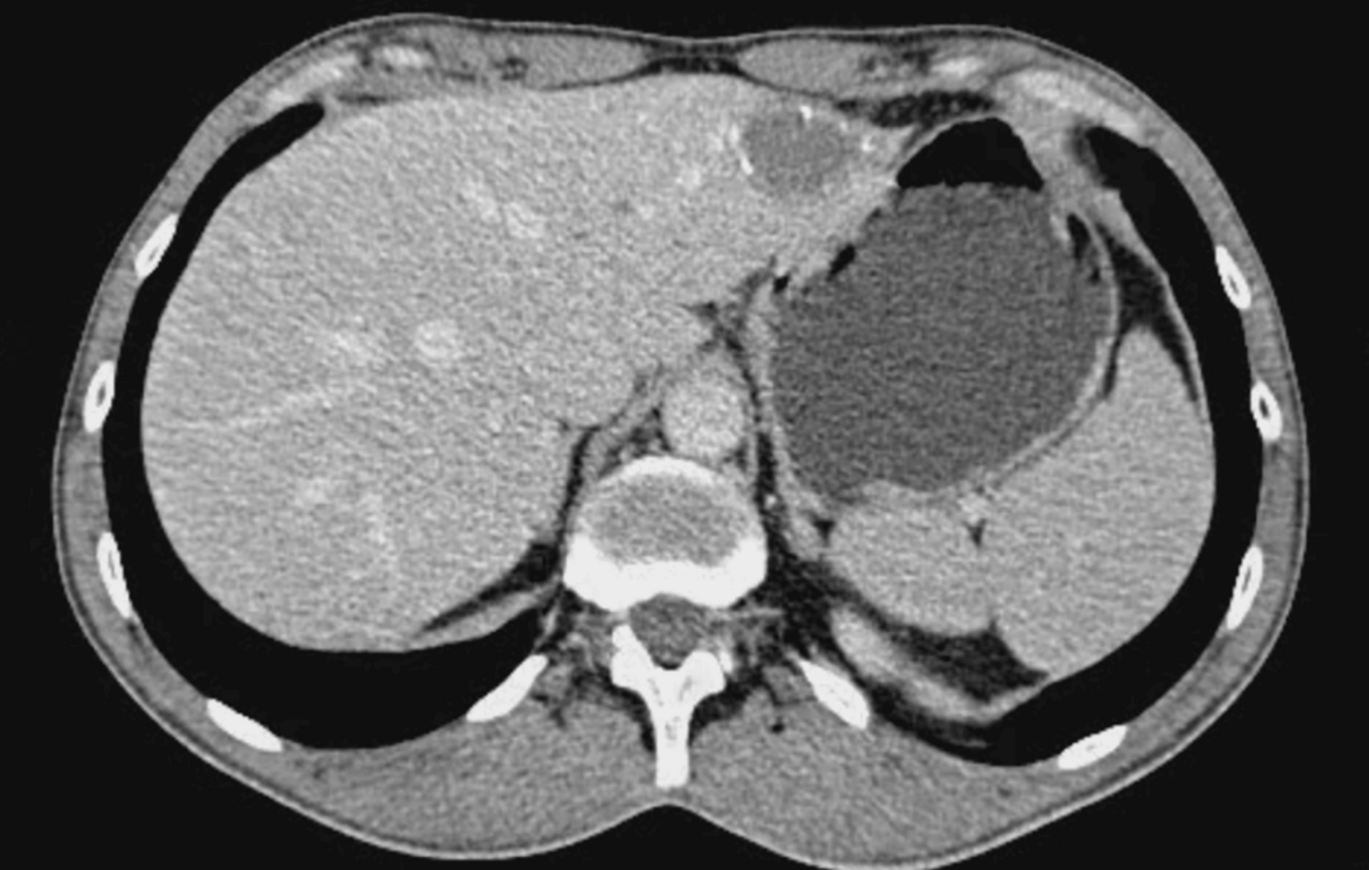
Contrast-enhanced CT demonstrating partial radiological response after nivolumab therapy Abdominal CT image showing a marked reduction in the size of the hepatic lesion in the left lobe (segments II–III) following systemic treatment with nivolumab, consistent with a sustained partial radiological response compared to baseline imaging.

Following a multidisciplinary discussion, the patient started systemic immunotherapy with nivolumab. The patient initially received four cycles every three weeks, followed by continued treatment over a four-year period. The patient remained clinically stable throughout treatment, with no significant immune-related adverse events. Subsequent imaging showed a sustained partial response with a significant reduction in the liver tumor (Figure 2).

Given the sustained radiological response, absence of extrahepatic disease, and resectability of the lesion, the case was re-evaluated in a multidisciplinary setting, and surgical resection was considered appropriate with curative intent. The patient underwent an open left sectionectomy (liver segments II and III). The patient recovered well from surgery and was discharged on postoperative day 2.

**Figure 3:**
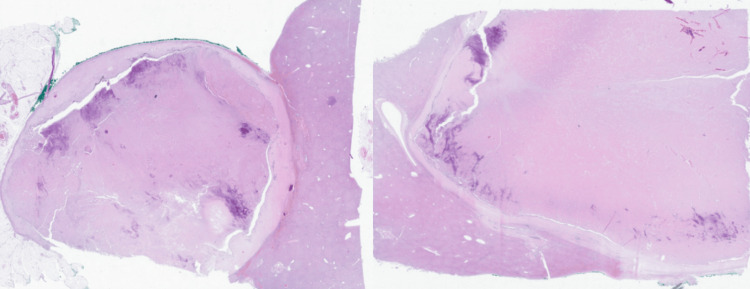
Histopathological findings demonstrating complete pathological response after hepatic resection Histological sections of the resected left liver specimen (hematoxylin and eosin staining) showing extensive areas of coagulative necrosis and fibrosis, associated with lymphoplasmacytic infiltrates and granulomatous inflammation, with no evidence of viable melanoma cells, consistent with a complete pathological response.

The surgical specimen underwent thorough pathological examination to assess the response to systemic therapy. Macroscopically, the resected left lateral section contained multiple well-circumscribed necrotic nodules without viable tumor. Histopathological analysis confirmed a complete pathological response, revealing no residual melanoma cells. Instead, extensive areas of coagulative necrosis and fibrosis were observed, accompanied by lymphoplasmacytic infiltrates, multinucleated giant cells, and granulomatous inflammation (Figure 3).

Further histological evaluation of the non-tumorous liver demonstrated chronic necrotizing granulomatous hepatitis, characterized by epithelioid granulomas and central necrosis, with no evidence of viable metastatic melanoma. Immunohistochemistry (HMB-45, S100, and Melan-A) consistently confirmed the absence of tumor cells. All resection margins were negative (R0).

**Figure 4:**
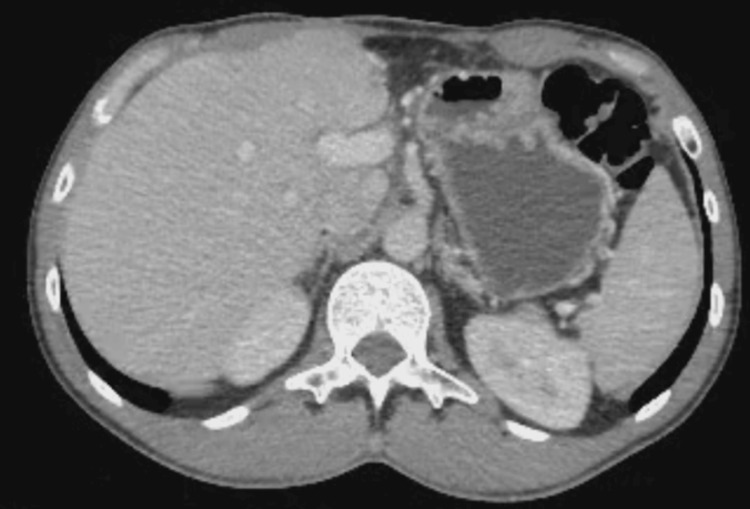
Follow-up contrast-enhanced CT after hepatic resection and immunotherapy Abdominal contrast-enhanced CT scan performed during follow-up after hepatic resection and nivolumab therapy, demonstrating no radiological evidence of residual or recurrent hepatic metastatic disease.

**Figure 5:**
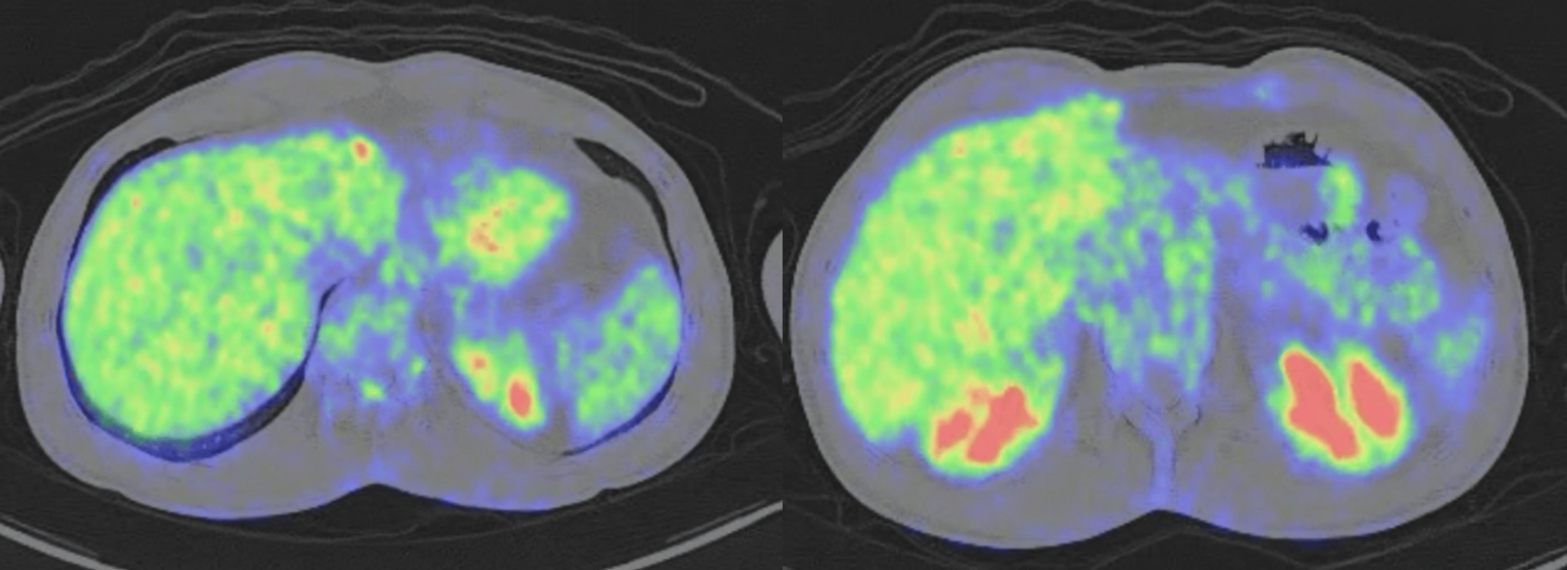
Follow-up FDG PET/CT after hepatic resection and immunotherapy Post-treatment fluorodeoxyglucose (FDG) PET/CT scan performed during follow-up after hepatic resection and nivolumab therapy, demonstrating no abnormal hypermetabolic activity suggestive of residual or recurrent metastatic disease. PET/CT: Positron emission tomography/computed tomography.

Given the complete pathological response, postoperative systemic therapy was not resumed, and no further cycles of nivolumab were administered after surgery. Serial follow-up imaging (Figures 4, 5) consistently confirmed the sustained absence of disease. Laboratory values progressively normalized, with lactate dehydrogenase and liver function tests returning to baseline.

At the most recent follow-up, 48 months after the initiation of nivolumab and 12 months after hepatic resection, the patient remained alive and disease-free, with no clinical, biochemical, or radiological evidence of recurrence.

## Discussion

Melanoma is an aggressive malignancy with a marked tendency for distant dissemination and historically poor outcomes once metastatic disease develops. Earlier systemic treatments, including chemotherapy and radiotherapy, offered limited benefit and were associated with significant toxicity. Over the last decade, the introduction of targeted therapies and immune checkpoint inhibitors has substantially improved the management and prognosis of patients with advanced melanoma [1,7].

Among these therapies, inhibitors of the programmed death-1 (PD-1) pathway, such as nivolumab, have become a key component of treatment, with the potential to induce durable responses in a subset of patients with metastatic disease [8,9]. However, responses are variable, and the optimal management of residual disease remains uncertain.

The liver is a recognized site of melanoma metastasis and is typically associated with an unfavorable prognosis. In selected patients, surgical resection or locoregional therapies may be considered, particularly within a multidisciplinary approach and in the setting of controlled disease [2-4,6].

Immunotherapy has been shown to improve survival in patients with unresectable advanced melanoma, with median overall survival increasing from approximately six months to nearly six years [9,10].

In the present case, the patient achieved a sustained radiological response to nivolumab, with no evidence of extrahepatic disease, allowing consideration of surgical resection. The finding of a complete pathological response following hepatectomy suggests that, in selected cases, surgery may be associated with favorable outcomes after systemic therapy. However, the contribution of surgery relative to ongoing systemic therapy alone cannot be determined in this context. Continuation of immunotherapy without surgical intervention could have represented an alternative management strategy. These findings should be interpreted with caution. As a single case report, this observation is subject to inherent limitations, including the inability to establish causality and the potential influence of patient selection.

Further studies are required to better define the role of metastasectomy in the immunotherapy era, including appropriate patient selection and timing of surgical intervention.

## Conclusions

Immune checkpoint inhibitors have improved the management of advanced melanoma and are widely used in both neoadjuvant and adjuvant settings. This case illustrates that, in selected patients with resectable hepatic disease and sustained response to immunotherapy, surgical resection may be considered as part of a multidisciplinary treatment strategy. However, the relative contribution of surgery compared with continued systemic therapy alone cannot be determined from a single case. Multidisciplinary evaluation remains essential to optimize management in the immunotherapy era.
